# The −842G/C Polymorphisms of PIN1 Contributes to Cancer Risk: A Meta-Analysis of 10 Case-Control Studies

**DOI:** 10.1371/journal.pone.0071516

**Published:** 2013-08-27

**Authors:** Hui-Rong Xu, Zhong-Fa Xu, Yan-Lai Sun, Jian-Jun Han, Zeng-Jun Li

**Affiliations:** Department of General Surgery, Shandong Cancer Hospital and Institute, Shandong Academy of Medical Science, Jinan, Shandong, China; The University of Texas MD Anderson Cancer Center, United States of America

## Abstract

**Background:**

Peptidyl-prolyl cis–trans isomerase NIMA-interacting 1 (PIN1) plays an important role in cancer development. The relationship between PIN1 −842G/C (rs2233678) polymorphism and cancer risk was inconclusive according to published literature.

**Methodology/Principal Findings:**

A literature search, up to February 2013, was carried out using PubMed, EMBASE and the China National Knowledge Infrastructure (CNKI) database. A total of 10 case-control studies including 4619 cases and 4661 controls contributed to the quantitative analysis. Odds ratio (OR) and 95% confidence intervals (95% CI) were used to assess the strength of association. Overall, individuals with the variant CG (OR = 0.728, 95% CI: 0.585,0.906; P_heterogeneity_<0.01) and CG/CC (OR = 0.731, 95% CI: 0.602,0.888; P_heterogeneity_<0.01) genotypes were associated with a significantly reduced cancer risk compared with those with wild GG genotype. Sub-group analysis revealed that the variant CG (OR = 0.635, 95% CI: 0.548,0.735; P_heterogeneity_ = 0.240) and CG/CC (OR = 0.645, 95% CI: 0.559,0.744, Pheterogeneity = 0.258) genotypes still showed an reduced risk of cancer in Asians; while no significant association was observed in Caucasians (CG vs.GG: OR = 0.926, 95% CI: 0.572,1.499, P_heterogeneity_<0.01; CG/CC vs. GG: OR = 0.892, 95% CI: 0.589,1.353; P_heterogeneity_<0.01). Furthermore, sensitivity analysis confirmed the stability of results. Begg's funnel plot and Egger's test did not reveal any publication bias.

**Conclusions:**

This meta-analysis suggests that the PIN1 −842G/C polymorphism is associated with a significantly reduced risk of cancer, especially in Asian populations.

## Introduction

Pro-directed phosphorylation is a critical signaling mechanism in various cellular processes, including transcription, RNA processing, cell cycle progression, cell proliferation and differentiation [1]–[3]. It has been demonstrated that the deregulation of this mechanism can lead to cell transformation and tumorigenesis [3], [4]. Peptidyl-prolyl cis–trans isomerase NIMA-interacting 1(PIN1), which belongs to the evolutionarily conserved peptidyl-prolyl isomerase (PPIase) family, is a 18 kDa protein containing a carboxy-terminal catalytic domain and a WW amino-terminal protein–protein interaction domain which can change conformation of phosphoproteins by recognizing and binding to specific phospho-Ser/Thr-Pro motifs [4], [5]. It has been demonstrated that PIN1 is associated with different signaling pathways such as cell-cycle progression, cellular proliferation, as well as neoplastic transformation [6], [7]. Previous studies have shown that PIN1 was overexpressed in a variety of human cancers [8], [9]. Further, its expression levels parallel the malignant properties in several types of cancer, such as lung cancer, colon cancer, breast cancer, prostate cancer, and oral squamous cell carcinoma [10]–[13]. These findings suggest that PIN1 may play an important role in cancer development.

The gene that encodes PIN1 protein is mapped to chromosome 19p13.2. Several studies have investigated the relationship between the single nucleotide polymorphisms (SNP, −842G/C, rs2233678) in the PIN1 promoter region and risk of cancers, such as breast cancer [14], [15], lung cancer [16], esophageal carcinoma [17], hepatocellular carcinoma [18], nasopharyngeal carcinoma [19], laryngeal squamous cell carcinoma [20], and squamous cell carcinoma of the head and neck [21]. However, these studies yielded different or even controversial results.

To confirm the association between −842(G>C) polymorphisms of PIN1 gene and cancer risk, we performed this meta-analysis by pooling all eligible studies to calculate the estimate of overall cancer risk and evaluate influence of cancer types and ethnicity.

## Methods

### Identification of Studies

A literature search was carried out using PubMed, EMBASE and China National Knowledge Infrastructure (CNKI) database up to February 2013. There was no restriction of origin or languages. Search terms included: “PIN1” or “rs2233678” in combination with “polymorphism” or “variant” and “cancer” or “neoplasm” or “malignancy”. The reference lists of each comparative study and previous reviews were manually examined to identify additional relevant studies.

### Inclusion and exclusion criteria

Studies were selected according to the following inclusion criteria: (1) case-control studies; (2) investigating the association between PIN1 rs2233678 (G>C) polymorphism and cancer risks; (3)cancers diagnosed by histopathology; (4) providing detail genotype frequencies. Studies without detail genotype frequencies were excluded. Titles and abstracts of searching results were screened and full text papers were further evaluated to confirm eligibility. Two reviewers(XH and XZ) independently selected eligible studies. Disagreement between the two reviewers was settled by discussing with the third reviewer (LZ).

### Data extraction

The following data was collected by two reviewers(XH and XZ) independently using a purpose-designed form: name of first author, publishing time, country where the study was conducted, genotyping methods, ethnicity, cancer types, source of control, number of cases and controls, genotype frequency in cases and controls. Different ethnicity descents were categorized as Asian and Caucasian. Cancer types were classified as breast cancer, squamous cancer (squamous cell carcinoma of head and neck, and laryngeal squamous cell carcinoma), and other cancers (nasopharyngeal carcinoma, esophageal carcinoma, lung cancer, and hepatocellular carcinoma). Eligible studies were defined as hospital-based (HB) and population-based (PB) according to the control source.

### Methodological quality assessment

The quality of eligible studies was evaluated by three reviewers (XH, XZ and LZ) independently by scoring according to a “methodological quality assessment scale” (*see Table S2: Scale for methodological quality assessment*), which was modified form a previous meta-analysis [22]. In the scale, 6 items were assessed, namely the representativeness of cases, the source of controls, ascertainment of relevant cancer, sample size, quality control of genotyping methods, and Hardy-Weinberg equilibrium (HWE). Quality scores ranged from 0 to 10 and a high score indicated good quality of the study. Three reviewers solved disagreement by discussion.

### Statistical analysis

The association strength between −842G>C (rs2233678) polymorphism and cancer risks was measured by odds ratio (OR) with 95% confidence intervals (95% CI). The estimates of pooled ORs were achieved by calculating a weighted average of OR from each study. A 95% CI was used for statistical significance test and a 95% CI without 1 for OR indicating a significantly increased or reduced cancer risk. The pooled ORs were calculated for homozygote comparison (CC versus GG), heterozygote comparison (GC versus GG), dominant (GC/CC versus GG) and recessive (CC versus GC/GG) models, assuming dominant and recessive effects of the variant G allele, respectively. Subgroup analyses were performed according to (i) cancer types, (ii) ethnicities, and (iii) source of control, to examine the impact of these factors on the association. To test the robustness of the association and characterize possible sources of statistical heterogeneity, sensitivity analysis was carried out by excluding studies one-by-one and analyzing the homogeneity and effect size for all of rest studies.

Chi-square based Q test was used to check the statistical heterogeneity between studies, and the heterogeneity was considered significant when p<0.10 [23]. The fixed-effects model (based on Mantel-Haenszel method) and random-effects model (based on DerSimonian-Laird method) were used to pool the data from different studies. The fixed-effects model was used when there was no significant heterogeneity; otherwise, the random-effects model was applied [24]. The between studies variance (τ^2^) was used to quantify the degree of heterogeneity between studies and the percentage of τ^2^ was used to describe the extent of heterogeneity explained [25]. Publication bias was assessed using Begg and Mazumdar adjusted rank correlation test and the Egger regression asymmetry test [26], [27].

HWE (Hardy-Weinberg equilibrium) was tested by Pearson's X^2^ test (P<0.05 means deviated from HWE). All analyses were performed using Stata version 11.0 (StataCorp, College Station, TX).

## Results

### Search results and characteristics of studies included in the meta-analysis

**Figure 1:**
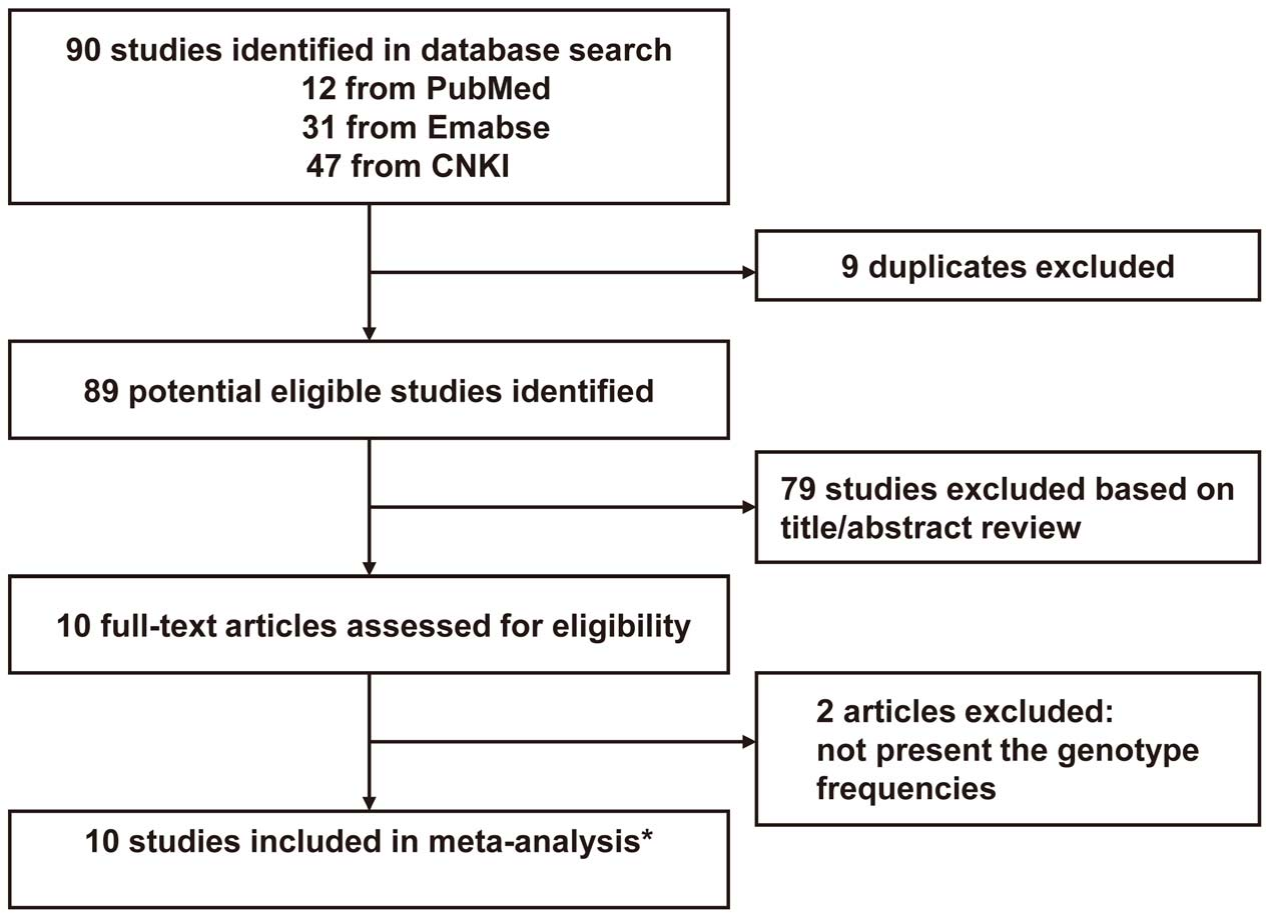
Flow diagram of the study selection process. In the study reported by Naidu R [15], three different populations (Malay, Chinese, and Indian) were included , and each of them was considered as a separate study in this meta-analysis.

The flow diagram of study identification is shown in Figure 1. A total of 90 citations were identified during the initial search. After the primary screening of titles and abstracts, we identified 10 papers. After detailed evaluation, two studies were excluded for not present the genotype frequencies. In the study reported by Naidu R and colleagues [15], participants were recruited from three different populations (Malay, Chinese, and Indian), and the genotype frequencies were presented separately, thus each of them was considered as a separate study in this meta-analysis. At last, 10 case-control studies [14], [15], [16], [17], [18], [19], [20], [21], including 4619 cancer cases and 4661 controls, assessing the association between −842(G>C) polymorphism of PIN1 and cancer risk, published between 2007 and 2013 were included in the meta-analysis (Baseline data and other details are shown in Table 1). Of them, seven studies were conducted in Asia [15], [16], [17], [19], [20], two in United States of America [14], [21], and remaining one in Europe [18]. Cancer cases were diagnosed histologically or pathologically in all studies. Polymerase chain reaction-restriction fragment length polymorphism (PCR-RFLP) assay was used for genotyping in 9 studies [14], [15], [16], [17], [19], [20], [21]. However, the method for genotyping was not described in one study [18]. Blood sample was used for genotyping in all studies. Genotype distribution of controls in all studies was consistent with HWE, except for Segat L's study [18] on hepatocellular carcinoma (P = 0.07).

**Table 1:** Characteristics of studies included in the meta-analysis.

First Author	Year	Country	Ethnicity	Genotyping Method	Cases/Controls	Cancer Type	Control	Cases			Controls			HWE
								GG	GC	CC	GG	GC	CC	
You Y	2013	China	Asian	PCR-RFLP	699/729	EC	Population	621	75	3	607	114	8	Y
Lu Y	2012	China	Asian	PCR-RFLP	178/156	NC	Hospital	135	22	21	110	38	8	Y
Cao WP	2012	China	Asian	PCR-RFLP	95/100	LSCC	Hospital	87	8	0	74	23	3	Y
Lu J	2011	China	Asian	PCR-RFLP	1559/1679	LC	Hospital	1380	170	9	1396	271	12	Y
Naidu R	2011	Malaysia	Asian	PCR-RFLP	107/80	BC	Population	78	28	1	53	24	3	Y
Naidu R	2011	China	Asian	PCR-RFLP	219/111	BC	Population	163	54	2	72	35	4	Y
Naidu R	2011	India	Asian	PCR-RFLP	61/61	BC	Population	45	15	1	48	11	2	Y
Han CH	2010	USA	Caucasian	PCR-RFLP	467/488	BC	Hospital	358	101	8	336	143	9	Y
Lu J	2009	USA	Caucasian	PCR-RFLP	1006/1007	SCCHN	Hospital	838	159	9	794	202	11	Y
Segat L	2007	Italy	Caucasian	null	228/250	HC	null	167	59	2	203	40	7	N

BC: breast cancer; EC: esophageal carcinoma;HC: hepatocellular carcinoma;

LC: lung cancer; LSCC: laryngeal squamous cell carcinoma; NC: nasopharyngeal carcinoma;

SCCHN: squamous cell carcinoma of the head and neck.

PB: population-based; HB: hospital-based.

### Meta-analysis results

**Figure 2:**
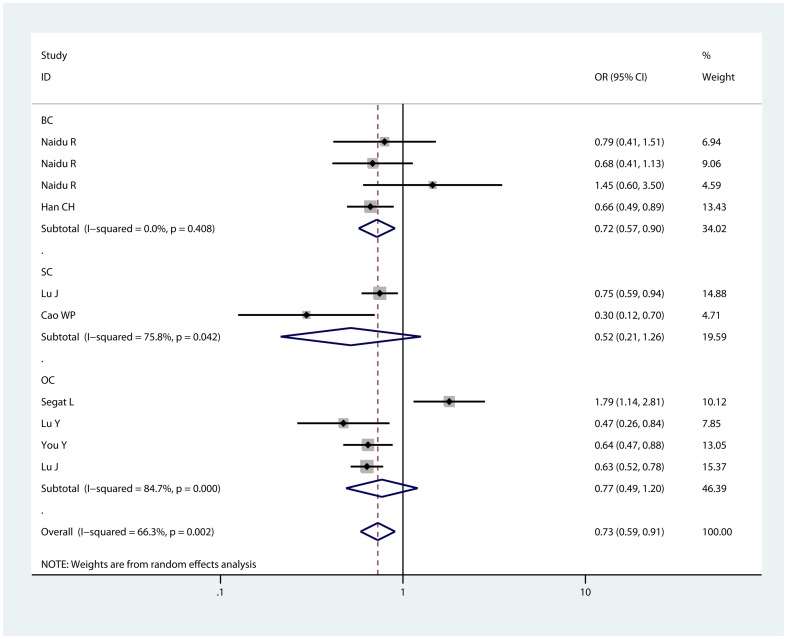
Forest plot of heterozygote comparison for overall comparison (GC vs. GG). BC: breast cancer; SC: squamous cancer; OC: other cancers.

**Figure 3:**
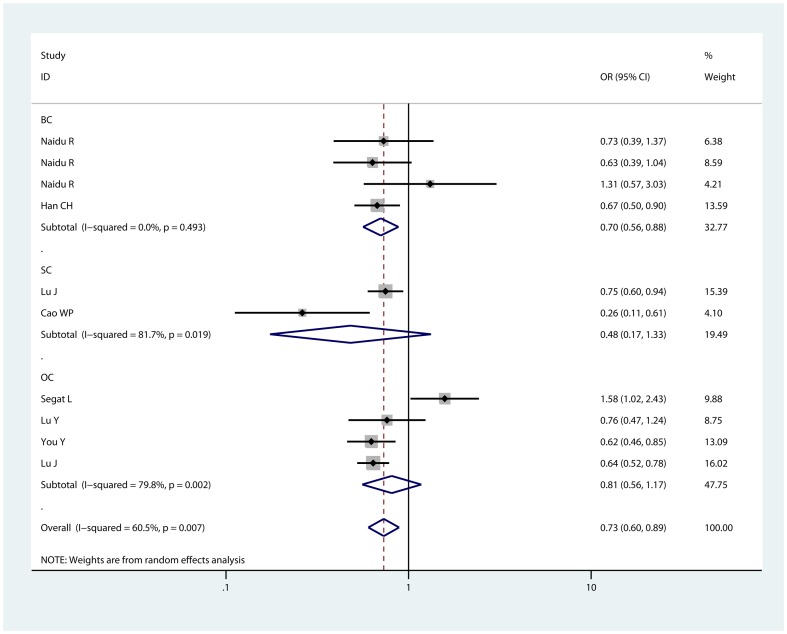
Forest plot of dominant model for overall comparison (GC/CC vs. GG). BC: breast cancer; SC: squamous cancer; OC: other cancers.

We observed a significantly reduced risk of cancer susceptibility in heterozygote comparison (CG vs GG: OR = 0.728, 95% CI: 0.585,0.906; P_heterogeneity_<0.01, Figure 2) and dominant model (CC/CG vs GG: OR = 0.731, 95% CI: 0.602, 0.888; P_heterogeneity_<0.01, Figure 3) when all eligible studies were pooled. The association strength between −842G/C polymorphisms in the PIN1 promoter region and cancer risk was shown in Table 2. As shown in Table 2, no significant association was found in homozygote comparison (CC vs GG: OR = 0.737, 95% CI: 0.513, 1.059; P_heterogeneity_ = 0.193) or recessive model (CC vs GG/CG: (,)OR = 0.653, 95% CI: 0.354, 1.203; P_heterogeneity_ = 0.088); however, a trend of reduced risk could be drawn.

**Table 2:** Stratified analyses of the −842G/C Polymorphisms in PIN1 Gene with cancer risk.

		CC vs GG		CG vs GG		CC/CG vs GG		CC vs GG/CG	
	N	OR	P_h_	OR	P_h_	OR	P_h_	OR	P_h_
Total	10	0.737(0.513,1.059)	0.193	0.728(0.585,0.906)*	0.002	0.731(0.602,0.888)*	0.007	0.653(0.354,1.203)	0.088
Cancer Types									
BC	4	0.532(0.255,1.108)	0.498	0.720(0.573, 0.905)*	0.408	0.705(0.564,0.881)*	0.493	0.576(0.278,1.197))	0.483
SC	2	0.613(0.270,1.393)	0.236	0.518(0.213, 1.256)	0.042	0.481(0.174,1.328)	0.019	0.658(0.289,1.498)	0.270
others	4	0.778(0.330,1.834)	0.065	0.766(0.487, 1.205)	<0.001	0.809(.0557,1.174)	0.002	0.741(0.172,3.182)	0.015
Ethnicities									
Asian	7	0.581(0.268,1.264)	0.077	0.635(0.548,0.735)*	0.240	0.645(0.559,0.744)*	0.258	0.515(0.173,1.530)	0.032
Caucasian	3	0.695(0.384,1.261)	0.627	0.926(0.572,1.499)	0.001	0.892(0.589,1.353)	0.004	0.725(0.401,1.310)	0.483
Source of Control									
PB	4	0.315(0.129,0.769)*	0.925	0.711(0.562,0.900)*	0.378	0.678(0.538,0.853)*	0.425	0.332(0.136,0.808)*	0.952
HB	5	0.974(0.639,1.486)	0.213	0.651(0.572,0.742)*	0.214	0.671(0.592,0.762)*	0.194	1.183(0.730,1.916)	0.125

N: number of studies included; OR: odds ratio; Ph: p value for heterogeneity; BC: breast cancer; SC: squamous cancer;

PB: population-based; HB: hospital-based;

* : OR with statistical significance.

We then performed sub-group analyses to investigate the effect of cancer types, ethnicity, and source of control. As for cancer types, increased cancer risk was found in the heterozygote comparison (CG vs GG: OR = 0.720, 95% CI: 0.573, 0.905; P_heterogeneity_ = 0.408) and dominant model(CC/CG vs GG: OR = 0.705, 95% CI: 0.564, 0.881; P_heterogeneity_ = 0.493) for breast cancer. In the sub-group analyses of squamous cancer, and other cancers, we did find any significant association between −842G/C polymorphisms in the PIN1 promoter region and cancer risk. As for hospital-based studies, we observed a significantly reduced risk of cancer susceptibility in homozygote comparison (CC vs GG: OR = 0.315, 95% CI: 0.129, 0.769; P_heterogeneity_ = 0.925), heterozygote comparison (CG vs GG: OR = 0.711, 95% CI: 0.562, 0.900; P_heterogeneity_ = 0.378), dominant model (CC/CG vs GG: OR = 0.678, 95% CI: 0.538, 0.853; P_heterogeneity_ = 0.425) and recessive model (CC vs GG/CG: OR = 0.332, 95% CI: 0.136, 0.808; P_heterogeneity_ = 0.952). However, for hospital-based studies, significant association between −842G/C polymorphisms in the PIN1 promoter region and reduced risks of cancers was found only in heterozygote comparison (CG vs GG: OR = 0.651, 95% CI: 0.572, 0.742; P_heterogeneity_ = 0.214), dominant model (CC/CG vs GG: OR = 0.671, 95% CI: 0.592, 0.762; P_heterogeneity_ = 0.194). Ethnicity, also, affected cancer susceptibility. In Asians, there was a statistically reduced cancer risk in the comparison of heterozygote (CG vs GG: OR = 0.635, 95% CI: 0.548, 0.735; P_heterogeneity_ = 0.240) and dominant model (CC/CG vs GG: OR = 0.645, 95% CI: 0.559, 0.744; P_heterogeneity_ = 0.258). Results for Asians were similar to that of overall comparisons of pooled eligible studies. In Caucasians, however, no significant association was found in each comparison. Taken together, these results revealed that −842G/C polymorphisms in the PIN1 promoter region was only associated with an increased risk of cancer in Asians.

### Heterogeneity between studies

Heterogeneity between studies in each comparison was shown in Table 2. After stratification, the heterogeneities decreased obviously in the subgroups of breast cancer, squamous cancer, the Asian population, Caucasian population, hospital-based controls, and population-based controls(P_heterogeneity_>0.1 in most genetic comparisons).

### Sensitivity analysis

Sensitivity analysis was performed to explore individual study's influence on the pooled results by deleting one single study each time from pooled analysis. The results showed that no individual study affected the pooled OR significantly (data not shown), since no substantial change was found.

### Publication Bias

**Figure 4:**
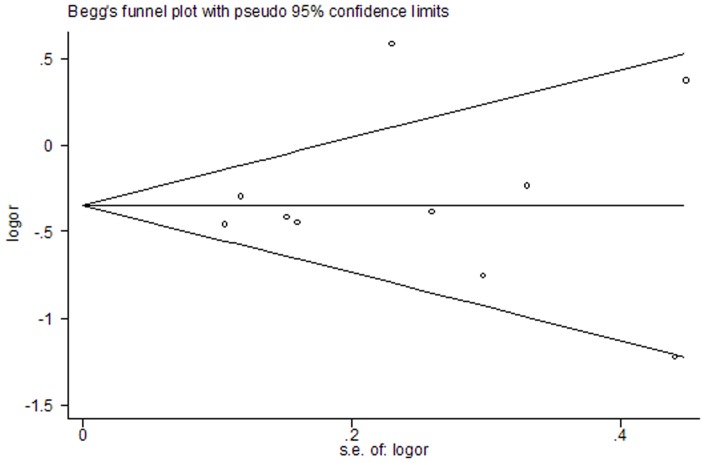
Funnel plot for publication bias. Heterozygous genetic model for overall comparison: GC vs GG. No publication bias was observed among studies using Begg's P value (P = 0.93) and Egger's (P = 0.73) test, which suggested there was no evidence of publication bias.

The potential publication bias of the literatures was .evaluated by funnel plot and Egger's test. No visual publication bias was found in the funnel plot (Figure 4). And Egger's test suggested that no publication bias was detected in all the comparison models (P>0.05).

## Discussion

In the present meta-analysis of 10 case-control studies, including 4619 cancer cases and 4661 controls, a significant association was found between PIN1 −842G/C polymorphism and reduced cancer risk under the heterozygous and dominant genetic models. Under the homozygous and recessive genetic model, there was no significant association between PIN1 −842G/C polymorphism and cancer risk. Overall, a significant association exists between −842G/C polymorphisms in the PIN1 promoter region and cancer risk. This finding indicates that the genetic variant in PIN1 promoter region may crucially modify the susceptibility of cancers.

Although PIN1 is not an oncogene itself, it is able to potentiate the function of several oncogenic pathways depending on other oncogenes [9]. Numerous targets of PIN1 were often deregulated in cancer, such as p53 [28], [29], p73 [30], beta-catenin [12], [31], cyclin D [13], [32], [33], [34], cyclin E [35], RAF1 [36], erbB2 [37] , MYC [38], and interleukin-8 [39]. Lu J et al found that the change from G to C may cause loss of the known gene-binding site that may regulate the PIN1 expression, and thereby deregulate its target protein leading to cancer development [19]. Previous studies suggested that high expression of PIN1 is correlated with tumor development and poor prognosis [40], [41].

In stratified analysis by cancer site, we found that −842G/C polymorphism in the PIN1 promoter region was statistically related with reduced breast cancer risks. However, we did not observe any significant association between the genetic variant and the susceptibility of other cancers. However,there are only two studies [20], [21] investigating the association between −842G/C polymorphism and squamous cell carcinoma risk, and only one study investigating the association between −842G/C polymorphism and risk of other cancers, including lung cancer [16], esophageal carcinoma [17],hepatocellular carcinoma [18], nasopharyngeal carcinoma [19]. So we should treat the result with caution, and more original case-control studies are needed to further evaluate the association between −842G/C polymorphism and different cancer types.

In the sub-group analysis of ethnicity, we found a significant association between −842G/C polymorphism and reduced risk of cancer in Asians but not in Caucasians. The different cancer risks in Asians and Caucasians were also reported in other meta-analyses [22], [42]. It is possible that different genetic backgrounds and the different environment they live in may account for these differences. As we know, different populations carry different genotype and/or allele frequencies of this locus polymorphism and may lead to various degrees of cancer susceptibility [43]. And different ethnic groups live with multiple life styles and environmental factors and thus yield diverse gene-environment interactions [44]. In addition, it was also likely that the relatively small sample size in Caucasians might cause the inconspicuousness.

During sub-group analyses, we found that control source also affected the association between −842G/C polymorphisms in the PIN1 promoter region and cancer risk. As for hospital-based studies, we observed a significantly reduced risk of cancer susceptibility in homozygote model, and recessive model. However, for hospital-based studies, no significant association between −842G/C polymorphisms in the PIN1 promoter region and risk of cancers was found in homozygote model, and recessive model. Further, it's worth noting that the majority (66.7%) studies of Caucasians use hospital-based controls, while most (57.1%) studies of Asians use population-based controls. So the ethnic interpretations are available to the inconsistency in control source stratification.

Some limitations might be included in the meta-analysis. First, we did not search for unpublished studies, so only published studies were included in our meta-analysis. Therefore, publication bias may have occurred although no publication bias was indicated from both visualization of the funnel plot and Egger's test. Second, the sample size of the included studies was not large enough, which could decrease the statistical power to better evaluate the association between −842G/C polymorphism in the PIN1 promoter region and cancer risk. Third, most of the included studies had conducted on Asians, and a few Caucasians. Thus, more samples should be collected from Caucasians.

In conclusion, this meta-analysis suggests that the −842G/C polymorphism in PIN1 gene is associated with a significantly reduced risk of cancer, especially in Asian populations. More well-designed studies focusing on different ethnicities and cancer types are warranted in the future.

## Supporting Information

Table S1 PRISMA Checklist.
(DOC)

Table S2 Scale for methodological quality assessment.
(DOC)
